# Identification of *DNAH6* mutations in infertile men with multiple morphological abnormalities of the sperm flagella

**DOI:** 10.1038/s41598-019-52436-7

**Published:** 2019-11-01

**Authors:** Chaofeng Tu, Hongchuan Nie, Lanlan Meng, Shimin Yuan, Wenbin He, Aixiang Luo, Haiyu Li, Wen Li, Juan Du, Guangxiu Lu, Ge Lin, Yue-Qiu Tan

**Affiliations:** 1 Institute of Reproductive and Stem Cell Engineering, School of Basic Medical Science, Central South University, Changsha, Hunan 410078 China; 2 Reproductive and Genetic Hospital of CITIC-Xiangya, Changsha, Hunan 410078 China; 3 National Engineering and Research Center of Human Stem Cell, Changsha, Hunan 410078 China

**Keywords:** Genetic testing, Infertility

## Abstract

Male infertility due to spermatogenesis defects affects millions of men worldwide. However, the genetic etiology of the vast majority remains unclear. Here we describe three men with primary infertility due to multiple morphological abnormalities of the sperm flagella (MMAF) from two unrelated Han Chinese families. We performed whole-exome sequencing (WES) and Sanger sequencing on the proband of family 1, and found that he carried novel compound heterozygous missense mutations in dynein axonemal heavy chain 6 (*DNAH6*) that resulted in the substitution of a conserved amino acid residue and co-segregated with the MMAF phenotype in this family. Papanicolaou staining and transmission electron microscopy analysis revealed morphological and ultrastructural abnormalities in the sperm flagella in carriers of these genetic variants. Immunostaining experiments showed that DNAH6 was localized in the sperm tail. This is the first report identifying novel recessive mutations in *DNAH6* as a cause of MMAF. These findings expand the spectrum of known MMAF mutations and phenotypes and provide information that can be useful for genetic and reproductive counseling of MMAF patients.

## Introduction

Infertility is a major health concern that affects more than 20 million men worldwide^1^. Factors contributing to male infertility include genetic disorders, urogenital infections, and immunological or hormonal abnormalities. Male infertility caused by sperm flagellar defects usually present with asthenozoospermia and teratozoospermia, which can also be observed in men with primary ciliary dyskinesia (PCD), a multisystem disorder caused by dysfunction of motile cilium and flagellum, leading to chronic rhinosinusitis, bronchiectasis, or heterotaxis^2^. However, some patients show similar sperm abnormalities but no other PCD manifestations, a condition known as multiple morphological abnormalities of the sperm flagella (MMAF)^3–8^.

MMAF is a characteristic form of severe asthenozoospermia defined by the presence of spermatozoa in the ejaculate with mosaic morphological abnormalities of the flagella—e.g., absent, short, bent, coiled, and irregular flagella^6,9^. Up to 20% of MMAF cases have a genetic origin^10^. However, to date only a few genes related to spermatogenesis or ciliogenesis—including *DNAH1*, *AKAP4*, *CAFP43*, and *CAFP44*^5,6,8,11,12^—have been identified as causative factors of MMAF in humans. Thus, the genetic factors underlying most MMAF cases are unknown.

*DNAH6* contains 77 exons and encodes a 4158-amino acid protein that belongs to the dynein protein family^13,14^, which includes multiple microtubule-associated motor protein complexes and plays an important role in ciliary movement and cell division^15^. *DNAH6* mutations were recently identified in a heterogeneous group of heterotaxy patients with abnormalities in respiratory tract cilia, which contributed to the development of PCD^16^. Additionally, *DNAH6* mutations were shown to be responsible for male infertility and premature ovarian insufficiency in humans^17–19^. Variants of *DNAH6* are also associated with changes in lung function in cystic fibrosis patients^20^. Different *DNAH6* mutations lead to distinct phenotypes; therefore, it is possible that they can contribute to the development of MMAF without PCD manifestations.

To evaluate this possibility, in this study, we examined 10 individuals with MMAF without PCD manifestations. We first identified novel compound heterozygous mutations in *DNAH6* in three infertile men with MMAF.

## Results

### Clinical data

**Figure 1:**
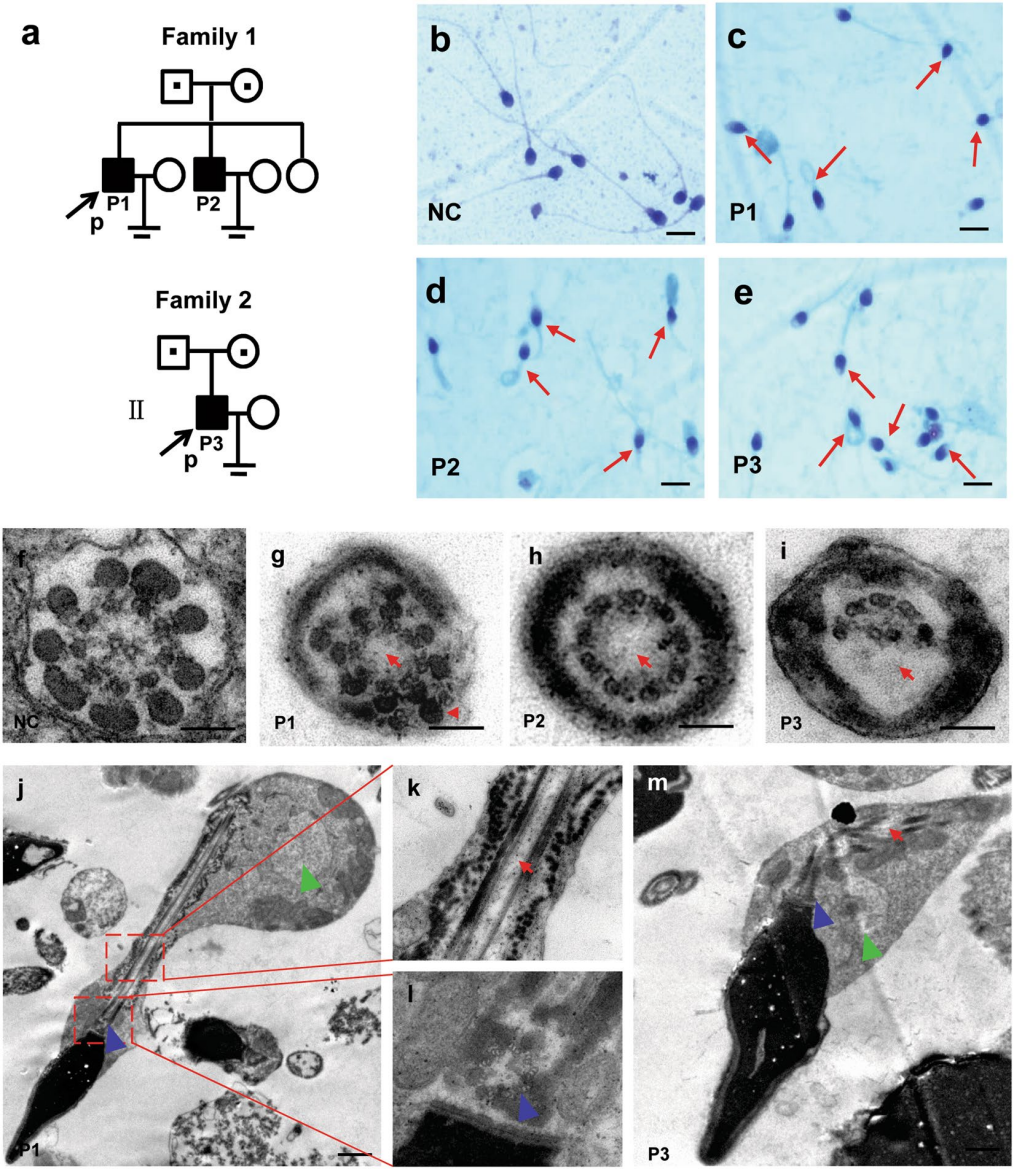
Pedigree of the two families analyzed in this study, and morphological and ultrastructural analysis of patient spermatozoa. (**a**) Filled and open symbols indicate the affected individuals and their unaffected relatives, respectively. A dot in the middle of a symbol indicates a heterozygous carrier. Probands are indicated with black arrows. (**b–e**) Papanicolaou staining of spermatozoa smears from the seminal fluid of the normal control (NC) (**b**) and the three patients (P1, P2, and P3) (**c**–**e**). Morphological abnormalities of sperm flagella were observed (red arrows), including absent, short, bent, coiled, and irregular flagella. Scale bars = 50 μm. (**f**–**m**) Ultrastructure analysis of spermatozoa obtained from the NC and patients by TEM. (**f**–**i**) Axonemal cross-sections of sperm flagella in NC, P1, P2, and P3. Absence of central microtubules and peripheral microtubule doublets (red arrow) or disorganization of outer dense fibers (red arrowhead) were observed. (**j**–**m**) Longitudinal sections of sperm flagella in NC, P1, and P2 showed that the disorganized fibrous sheath or mitochondrial sheath, and a lack of axonemal CPC in the sperm flagella of patients, and tails with a cytoplasmic mass were shown (green arrowhead). The sperm neck area including proximal centriole, striated column, vault (blue arrowhead) are visible. Scale bars = 0.1 μm.

Routine semen and sperm morphology analyses were carried out for ten patients presenting with severe asthenozoospermia resulting from a combination of multiple morphological defects of the sperm flagella including: absent, short, bent, coiled or irregular width; eight had 100% immobile spermatozoa and two had sperm motility <10% (Fig. 1a–e and Table 1). None of the subjects showed any other PCD-associated symptoms. There were almost no spermatozoa (1.5%–6.0%) with normal morphology in the patients’ ejaculate; short, absent, and coiled flagella were the most frequently observed phenotypes (Table 1). The ultrastructure of patients’ spermatozoa (P1, P2, and P3) by transmission electron microscopy (TEM) frequently revealed absence of the central pair complex (CPC) of microtubules (Fig. 1f–m), other defects were occasionally seen, including peripheral microtubule doublets or disorganization of outer dense fibers (Fig. 1f–m). Longitudinal sections showed that the disorganized fibrous sheath or mitochondrial sheath, and a lack of axonemal CPC in the sperm flagella of patients, and tails with cytoplamic mass containing unassembled components of the sperm flagellum were frequently observed. Yet, the head-tail connection region of the sperm was morphologically normal, including 9 regularly arranged triplets, the closely attached striated column and the vault at the base body (Fig. 1f–m). Taken together, these three patients were diagnosed with the MMAF syndrome according to previously established criteria^6^.

**Table 1:** Semen characteristics and sperm morphology in the ten patients under light microscopy.

Semen parameters	P1^a^	P2^a^	P3^a^	P4^c^	P5^b^	P6^c^	P7^b^	P8^c^	P9^c^	P10^c^
Age	41	34	35	46	26	35	28	27	43	36
Volume (ml)	4.7	2.5	2.9	2.3	3.2	2.5	3.2	2.2	2.5	3.1
Concentration (10^6^ ml^−1^)	10.1	6.8	11.3	25.3	10.1	11.5	20.6	42.1	20.5	8.2
Progressive motility (%)	0	5.3	0	0	0	0	0	0	0	0
Motility (%)	0	8.8	0	0	0	0	3.5	0	0	0
Normal flagella (%)	2.4	6.0	2.7	2.6	3.7	1.8	2.3	4.7	1.5	3.0
Absent flagella (%)	21.0	19.0	22.3	18.2	23.1	20.2	16.7	16.3	13.5	24.0
Short flagella (%)	51.0	48.5	45.6	52.8	54.9	59.5	64.5	59.0	68.0	50.5
Coiled flagella (%)	12.5	17.5	14.4	12.5	10.3	9.5	6.5	8.5	7.5	12.5
Bent flagella (%)	5.1	3.0	5.5	5.5	2.0	3.5	4.5	5.0	4.5	3.0
Irregular width (%)	8.0	6.0	9.5	8.4	6.0	5.5	5.5	6.5	5.0	7.0
Affected spermatozoa (%)	97.6	94.0	97.3	97.4	96.3	98.2	97.7	95.3	98.5	97.0

Note: ^a^Represent that the genetic cause of these patients might be *DNAH6* mutations; ^b^represent that the genetic cause of these patients might be *DNAH1* mutations; ^c^represent that the genetic causes of these patients remain unknown.

### Identification of DNAH6 mutations by WES

We screened for genes potentially causing MMAF in the proband (P1) by WES. We obtained 12.5 Gb of raw data with a mean depth of 159.28 folds for the target regions (Table S1). After mapping these data to the reference genome sequence (Hg 19), we identified 107,317 single nucleotide polymorphisms (SNPs) and 20,020 insertions/deletions (Indels) (Table S2). For rare inherited diseases, the frequency of possible pathogenic variants in the healthy population is very low. We filtered the WES results against a minor allele frequency >5% in publicly available SNP and Indel databases (Table S3); a total of 18,881 variants were retained, of which 362 were predicted to be deleterious. For infertile patients from a non-consanguineous family, homozygous or compound heterozygous variants are preferentially considered; based on these criteria, only five and eight variants, respectively, were retained. We focused on variants relevant to the phenotype in terms of expression and biological process, including model organisms with a male sterility phenotype similar to that observed in this family. Only one compound heterozygous variants of *DNAH6* (NM_001370.1: c.6582 C > A, p. D2194E and c.11258 G > A, p. G3753D) satisfied these criteria (Table S4). After reviewing the literature, we concluded that these were most likely to be the disease-causing mutations.

**Figure 2:**
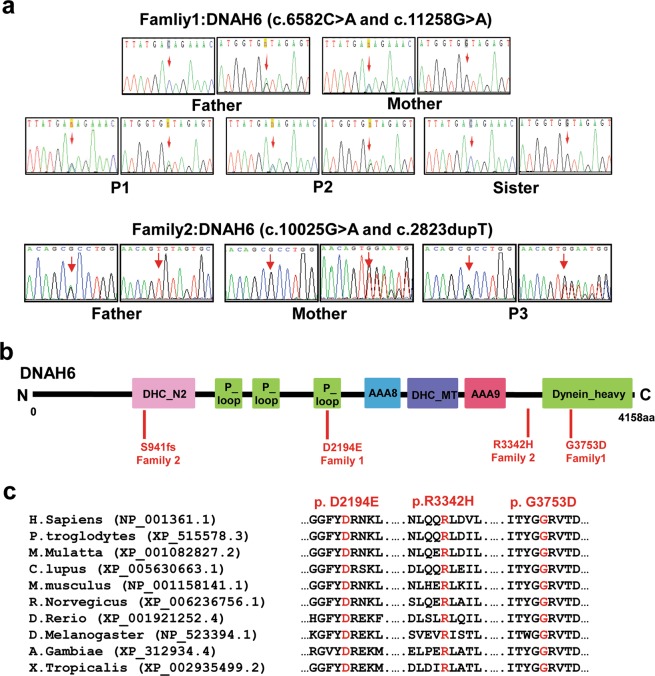
Segregation analysis and functional characterization of the *DNAH6* mutation. (**a**) Sequence chromatograms for *DNAH6* (c.6582 C > A and c.11258 G > A) in family 1, and *DNAH6* (c.10025 G > A and c.2823dupT) in family 2. Both unaffected parents were heterozygous, whereas the patients (P1, P2, and P3) were compound heterozygous for these mutations and the unaffected sister harbored the wild-type variant. (**b**) Structure of DNAH6 protein; predicted functional domains are shown together with the position of novel mutations identified in MMAF patients. (**c**) Cross-species alignment showing strong conservation at positions 2194 and 3753 in *DNAH6*.

We then performed Sanger sequencing and co-segregation analysis using the available DNA samples from family 1. The results showed that the affected brothers (P1 and P2) had the same compound heterozygous mutations (c.6582 C > A and c.11258 G > A) in *DNAH6* (Fig. 2a and Table S5); their father carried one heterozygous variant (c.6582 C > A), while their mother carried the other (c.11258 G > A). Their unaffected sister (II-3) harbored the wild-type allele. The mode of inheritance was consistent with an autosomal recessive mode of inheritance.

To validate our observations, we screened for *DNAH6* mutations in another eight patients with MMAF (P3–P10) by WES. We detected another *DNAH6* compound heterozygous mutation in P3 (family 2). Sanger sequencing confirmed that P3 harbored the compound heterozygous mutations c.10025 G > A and c.2823dupT in *DNAH6*, and segregation analysis demonstrated that these were inherited from the unaffected father (I-1) and mother (I-2), respectively (Fig. 2a). These *DNAH6* mutations were not detected in the remaining seven patients. Notably, two of the seven patients (P5 and P7) were found to have novel mutations in the known MMAF gene *DNAH1*(NM_015512: c.G5105C: p.R1702P and c.11726_11727del: p.P3909fs for P5; c.8151-1 G > C and c.C12286T:p.R4096C for P7) (Table S5), indicating that MMAF is a genetically heterogeneous disorder.

### Impact and expression profile of DNAH6 mutations

**Figure 3:**
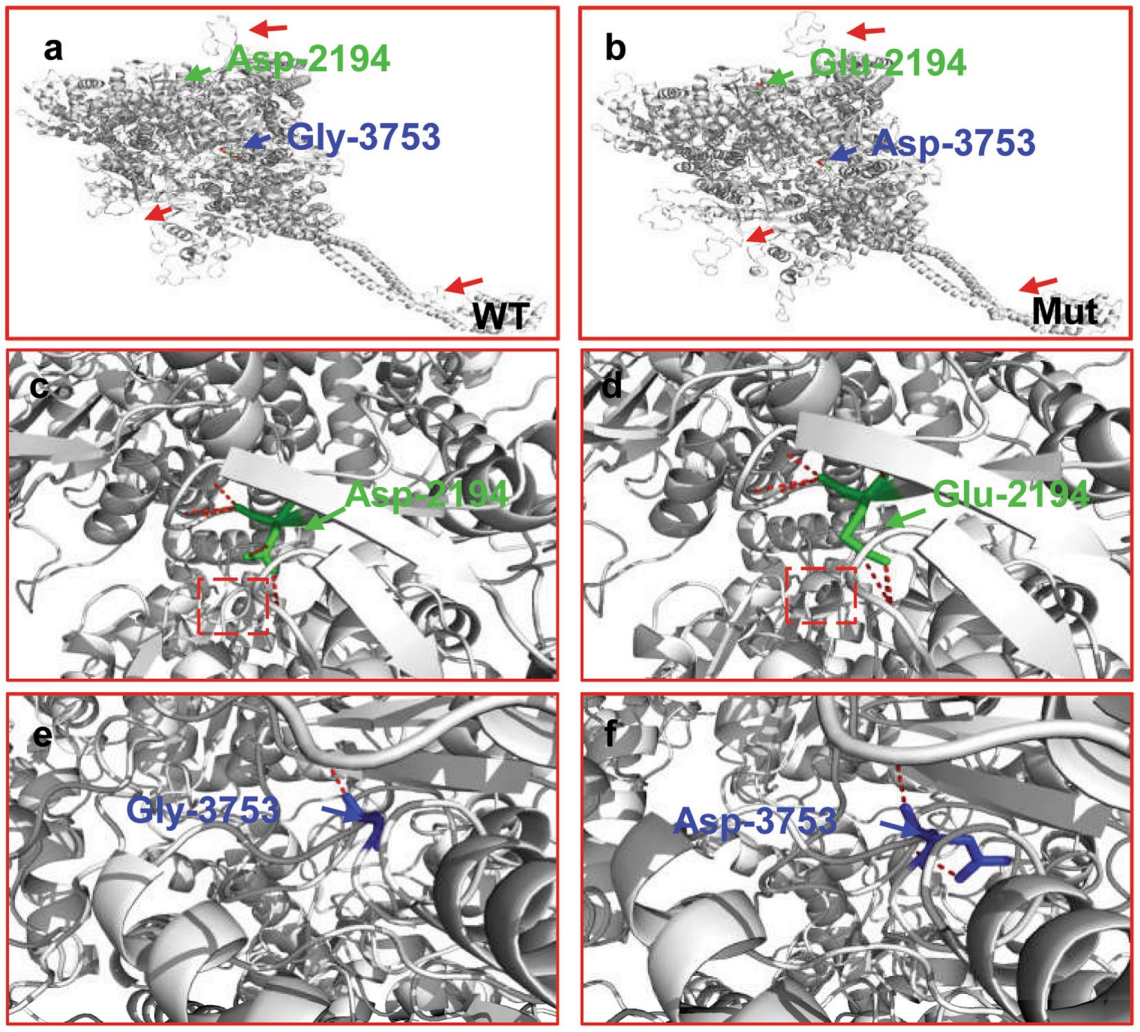
Modeling of wild-type and mutant DNAH6. Molecular structures of wild-type and mutant DNAH6 proteins were modeled with SWISS-MODEL software based on the template of dynein heavy chain, cytoplasmic (3vkh.pdb). (**a**,**b**) Structures of wild-type and mutant DNAH6 proteins; β-sheets (red arrows) are altered compared to the wild-type protein. (**c**,**d**) Enlarged view of the Asp-2194 site in wild-type and mutant DNAH6 protein. (**e**,**f**) Enlarged view of the Gly-3753 site in wild-type and mutant DNAH6 protein. Both Asp2194Glu and Gly3753Asp mutations of DNAH6 altered the structure of the protein. The positions of mutations are highlighted in blue.

DNAH6 variants identified in the unrelated patients were located on predicted functional domains (Fig. 2b) and resulted in the substitution of conserved residues (Fig. 2c). All of the mutations were predicted to be deleterious by in silico analysis (Table S5). The three-dimensional structure of DNAH6 (1006–4158 amino acid residues) was modeled to analyze the impact of *DNAH6* Asp2194Glu and p.Gly3753Asp in P1. The mutations changed part of the protein structure, including the β-sheets (Fig. 3a,b) and an α-helix (Fig. 3c,f), generating a misfolded protein with potentially reduced ATPase activity and microtubule-binding capacity.

**Figure 4:**
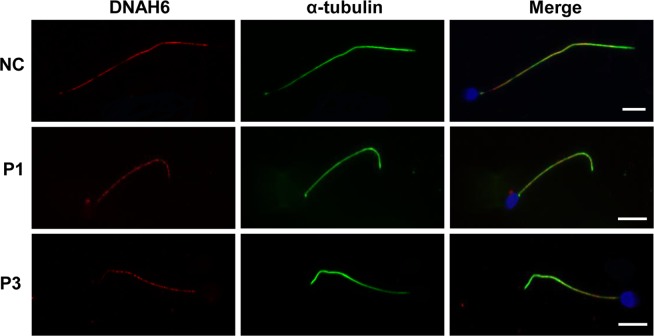
Immunolabeling of spermatozoa samples from *DNAH6*-mutant patients and normal controls (NC). Immunostaining of acetylated α-tubulin (green) and DNAH6 (red) in spermatozoa of NC and patients (P1 and P3). Nuclei were stained with 4′,6-diamidino-2-phenylindole (DAPI; blue). DNAH6 is specifically expressed in the sperm tail. Sperm tails in patients were short compared to those in the NC. Scale bars = 5 μm.

A reverse transcription PCR analysis showed that *DNAH6* transcript was highly expressed in ciliated tissues in both adult humans and mice including testis, ovary, brain, and lung, and was weakly expressed in human stomach and kidney tissues. No expression was detected in other tissues such as eye, heart, and liver (Fig. S1). We also determined the expression and location of DNAH6 protein in the spermatozoa of patients (P1 and P3) by immunofluorescence analysis (Fig. 4). DNAH6 were localized in the sperm tail of normal spermatozoa (Fig. 4), and the expression level of DNAH6 in patients’ spermatozoa was not changed compared to the normal controls (Fig. 4).

**Figure 5:**
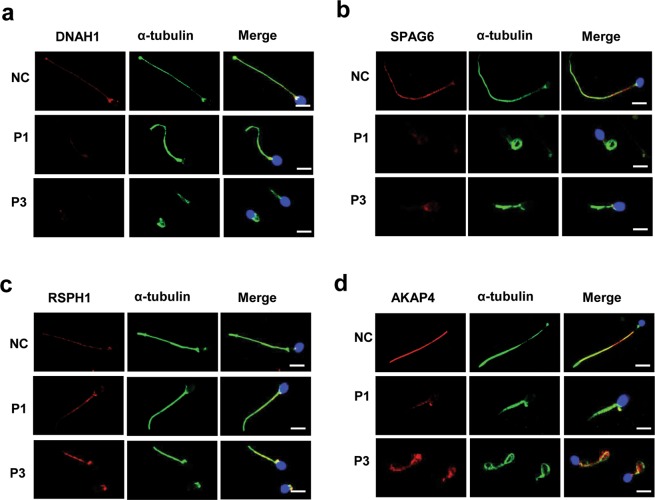
*DNAH6*-mutant patients have abnormal, disorganized sperm axonemes. (**a**,**b**) Spermatozoa from normal control (NC) and *DNAH6*-mutant patients (P1 and P3) were double-labeled with antibodies against acetylated α-tubulin to identify axonemes and basal bodies (green), DNAH1 as a marker of the inner dynein arm (red), and SPAG6 as marker of CPC (red). Both α-tubulin and DNAH1 or SPAG6 were colocalized along the full length of axonemes in sperm flagellum from the NC (upper panel). In contrast, in MMAF patients (P1 and P3) (lower panels), DNAH1 or SPAG6 expression was markedly reduced in the axonemes compared to NC. (**c**,**d**) Spermatozoa from control and *DNAH6*-mutant patients (P1 and P3) was double-labeled with antibodies against acetylated α-tubulin to identify axonemes and basal bodies (green), RSPH1 as a marker of the radial spoke (red), and AKAP4 as a marker of the fibrous sheath (red). Scale bars = 5 μm.

To characterize the ultrastructural defects observed by TEM at the molecular level, we performed immunostaining using antibodies targeting various axonemal proteins. In spermatozoa from *DNAH6*-mutant patients, expression of DNAH1 and SPAG6 (markers for the inner dynein arm and CPC, respectively) were remarkably reduced relative to the control (Fig. 5a,b). In patients with *DNAH6* mutations, expression levels of the radial spoke marker RSPH1 were comparable to that in the control (Fig. 5c), suggesting that the mutations do not affect the radial spokes of flagella. However, in *DNAH6*-mutant patients, the fibrous sheath marker AKAP4 showed abnormally diffuse expression all along the midpiece and principle piece (Fig. 5d), whereas a restricted staining in the principle piece was in the normal control, suggesting the affected location and disorganized assembly of fibrous sheath due to mutations in *DNAH6*. Taken together, these results suggest that *DNAH6* mutations cause severe axonemal or peri-axonemal disorganization, which might lead to the impair of sperm motility.

## Discussion

Infertility is a global medical and social problem with both physical and psychosocial consequences. Asthenozoospermia is particularly prevalent, occurring in ~19% of infertile men^21^; Generally, sever asthenozoospermia cases represent a heterogeneous group, and genetically-associated asthenozoospermia mainly includes PCD and MMAF^6,7,22^. In this study, we identified novel compound heterozygous mutations in the *DNAH6* gene in three patients with MMAF from two unrelated Chinese families. This is the first study reporting *DNAH6* as a recessive causative gene for this condition.

*DNAH6* encodes axonemal dynein heavy chain that functions as an inner dynein arm component^13^. Dyneins are ATP-fueled motor proteins that generate force and movement on microtubules in a range of biological processes including ciliary beating, cell division, and intracellular transport^15^. Mutations in *DNAH6* have been shown to cause PCD as well as heterotaxy by disrupting the CPC of motile cilia^16^. *DNAH6* can act both recessively and possibly through trans-heterozygous interactions with other PCD genes such as *DNAH1* or *DNAH5*^16^. Another member of the dynein heavy chain family, DNAH1, is required in spermatozoa for the formation of inner dynein arms and is important for the assembly and biogenesis of the flagellar axoneme^6,23^. Mutations in *DNAH1* can cause MMAF without any other PCD-associated symptoms^6,23^. Considering their structural and functional similarities, we speculate that DNAH6 may also participate in axoneme assembly and flagellar motility. In the present study, we confirmed that DNAH6 was highly expressed in tissues harboring cilia in both adult humans and mice such as testis, ovary, brain, and lung. Moreover, both TEM analysis and immunostaining revealed that *DNAH6* mutations were associated with severe axonemal disorganization and markedly reduced DNAH1 expression. Additionally, similar to *DNAH1* mutation phenotypes, patients with *DNAH6* mutations in our study presented MMAF without other PCD-related symptoms as well as aberrant axonemal structure of sperm flagella, suggesting that DNAH6 plays distinct roles in axoneme regulation in somatic and germ cells. Dynein is involved in the process of spermatogenesis^24^; patients with a rare homozygous missense mutation in *DNAH6* exhibited azoospermia and oligozoospermic (one patient)^17^. These findings provide evidence that DNAH6 contributes to flagellar axoneme assembly during spermatogenesis.

Recently, compound heterozygous missense mutations (c.2454 A > T, p.E818D; c.7706 G > A, p.R2569H) in *DNAH6* were shown to cause sperm head anomalies (30% headless spermatozoa and 69% globozoospermia)^19^. These investigators found that DNAH6 was localized in the neck region of normal spermatozoa, and DNAH6 mRNA and protein were completely absent in carriers of the mutations. We carefully considered their findings, however, in our study, DNAH6 was detected in the sperm tail and ultrastructural analysis did not find obvious abnormality in sperm head or the neck region in patients with *DNAH6* mutations. We found that compound heterozygous missense mutations in *DNAH6* were associated with MMAF.

We identified novel compound heterozygous mutations of *DNAH6* in both patients with MMAF that were predicted to be pathogenic by in silico analysis. The mutated amino acids (Asp2194Glu and p.Gly3753Asp) in P1—located in the third P-loop and dynein heavy chain domains, respectively, of DNAH6—were shown to be highly conserved across species. The P-loop has nucleoside triphosphate hydrolase activity and binds and hydrolyzes ATP^25^, and is the main functional domain of the dynein protein family. The dynein heavy chain domain located in the C terminus of DNAH6 has ATPase activity and binds microtubules, and functions as a motor for the movement of organelles and vesicles along microtubule tracks^26,27^. The three-dimensional model of the protein suggested that the identified compound heterozygous mutations in *DNAH6* alters part of the protein structure, resulting in protein misfolding; this could reduce the ATPase activity and microtubule-binding ability of DNAH6. These results suggest that the amino acids at these positions are critical for the process of spermatogenesis. Although our identification of *DNAH6* mutations in unrelated patients with MMAF suggests a causative relationship, functional validation of these mutations and their effects on spermatogenesis require further investigation.

Intracytoplasmic sperm injection (ICSI) is an assisted reproductive technology that is an effective way to achieve pregnancy in cases where the male exhibits grossly abnormal semen parameters, including MMAF^10,28–31^. Patients with MMAF harboring *DNAH1* mutations have a good prognosis following ICSI, with 70.8% overall fertilization, 50.0% pregnancy, and 37.5% delivery rates^32^. In our study, ICSI using ejaculated live spermatozoa from the proband (P1) with MMAF resulted in the fertilization of 14/17 oocytes, with eight transferable embryos. Clinical pregnancy was confirmed after three embryos were transferred to his wife. However, no fetal heartbeat was detected by ultrasound and the pregnancy ended in abortion (data not shown). Therefore, whether successful ICSI outcomes can be achieved in MMAF patients with *DNAH6* mutations requires further investigation.

In conclusion, we identified novel compound heterozygous mutations in *DNAH6* that are likely responsible for the disrupted flagellation of spermatozoa in patients with MMAF. Our findings expand the spectrum of known *DNAH6* mutations and phenotypes and provide useful information for genetic and reproductive counseling of patients with MMAF.

## Methods

### Patients

Ten male patients aged 26–46 years (mean age: 35.0 years) of the Hand ethnic group from Hunan province in China were treated in the Reproductive and Genetic Hospital of CITIC-Xiangya for primary infertility. The proband P1 (41 years old) and P2 (34 years old) from one family (family 1) were brothers whose parents were not consanguineous (Fig. 1a). The sister of the proband (29 years old) lacked any observable symptoms but had not attempted to procreate; therefore, her fertility status was unclear. The other eight unrelated patients (P3–P10), whose parents were not consanguineous either, were not related to one another. All patients had a normal 46, XY karyotype, and no abnormalities were observed by Y chromosome microdeletion analysis. Other causes of infertility such as drugs and exposure to gonadotoxic factors were excluded. None of the patients reported any additional symptoms associated with PCD. The patients’ parents were all healthy and fertile.

The study protocol was approved by the Institutional Ethics Committees of Central South University and Reproductive Genetic Hospital of CITIC-Xiangya, China. Written, informed consent was obtained from all participants. All procedures were performed in accordance with approved guidelines.

### Semen and sperm morphological analyses

Semen samples were collected from each patient through masturbation after 3–5 days of sexual abstinence. Each semen parameter was measured at least three times according to the World Health Organization (WHO) 2010 guidelines^33^. Morphological abnormalities of the flagella were classified as (1) absent, (2) short, (3) coiled, (4) bent, or (5) irregular width^6^ as determined by Papanicolaou staining. The percentages of morphologically normal and abnormal spermatozoa were evaluated according to WHO guidelines.

### Transmission electron microscopy (TEM)

Seminal fluid samples from normal control subjects (fertile adult donors who provided written informed consent to participate in this study) and patients were fixed overnight with 2.5% glutaraldehyde (Sigma-Aldrich, St. Louis, MO, USA) in 0.1 M phosphate buffer (pH 7.4) followed by 1% osmium tetroxide for 2 h for TEM analysis, which was carried out as previously described^34^. The 1-μm-thick sections were stained with toluidine blue for light microscopy, and 70- to 90-nm-thick sections were contrasted with uranyl acetate and lead citrate and examined with an H7700 electron microscope (Hitachi, Tokyo, Japan). Digital images were captured using a MegaView III digital camera (EMSIS GmbH, Münster, Germany).

### WES and bioinformatic analysis

WES analysis was performed for samples from nine patients (P1 and P3–P10) and targeted Sanger sequencing was performed for P2, whose sample had not been available in time for inclusion in WES. Sequences were captured with the SureSelect v.4 platform (Agilent Technologies, Santa Clara, CA, USA) and the enriched library was sequenced with the Illumina HiSeq. 2000 system as previously described^35^. Raw reads after removing the adaptors were aligned to NCBI GRCh37 (Human reference genome Hg19) using the Burrows–Wheeler Aligner^36^, followed by the removal of PCR duplicates and sorting using Picard (http://broadinstitute.github.io/picard/). Variant identification was performed using GATK package^37^ according to the recommended best practices, including base recalibration variant calling with Haplotype Caller, quality score recalibration, and annotation using ANNOVAR software^38^.

We filtered the variants obtained by WES with minor allele frequencies of 5% in dbSNP, 1000 Genomes Project, or ExAC databases according to the following criteria: predicted to be deleterious; homozygous or compound heterozygous for the alternate allele; relevant phenotype based on comprehensive expression data (high expression in testis)^39^; the Gene Ontology term “biological process associated with spermatogenesis^40^”; and model organism data (male infertility phenotype observed in animal models)^41^.

### Sanger sequencing

Forward and reverse PCR primers targeting mutation regions in the *DNAH6* gene had the following sequences: c.6582 C > A: 5′-CCATCTTGTGCCCTGACAGT-3′ and 5′-GTCTGTCCACCCTCTGAAGC-3′; c.11258 G > A: 5′-GGAAGGAAATTCTGTTGTGTGCT-3′ and 5′-TGGGGATAGGGTGTGGCTATAA-3′; c.10025 G > A: 5′-CTGAATGCGAACAAGGGAGAC-3′ and 5′-AGATAGGGTTCCTTGCTGACG-3′; and c.2823dupT: 5′-TGAAAGGAATAAACAGGTGATGCT-3′ and 5′-ACTCTGAATGCCCCTCCTAAC-3′. These regions were amplified by PCR using Ex Taq DNA polymerase (Bio-Rad, Hercules, CA, USA) in all patients and their family members. PCR products were sequenced on a 3730XL system (Applied Biosystems, Foster City, CA, USA) according to the manufacturer’s instructions.

### Evolutionary conservation and in silico analyses

Evolutionary conservation analysis was performed by aligning the amino acid sequences of DNAH6 proteins from different vertebrate species obtained from the GenBank database (https://www.ncbi.nlm.nih.gov/homologene/). The potential pathogenicity of DNAH6 mutations was predicted by in silico analysis using MutationTaster (http://www.mutationtaster.org/), Sorting Intolerant From Tolerant (http://sift.jcvi.org/), Polyphen-2 (http://genetics.bwh.harvard.edu/pph2), and Combined Annotation Dependent Depletion (cadd.gs.washington.edu/) programs. Structural analysis of DNAH6 (NP_001361.1) variants in P1 was carried out using SWISS-MODEL software (https://swissmodel.expasy.org) based on the template of dynein heavy chain, cytoplasmic (3vkh.pdb). PyMol (http://www.pymol.org) was used to visualize protein structure.

### Expression analysis

Total RNA was extracted from tissues of fertile adult donors (who provided written informed consent for their participation in this study) and wild-type C57BL/6 adult mice using TRIzol reagent (Invitrogen, Carlsbad, CA, USA), and 1 μg was reverse transcribed into cDNA using a the GoScript Reverse Transcription System (Promega, Madison, WI, USA) according to the manufacturer’s instructions. To evaluate the expression analysis of human *DNAH6* and mouse *Dnah6* genes, PCR amplification was performed with 1 μl of cDNA. The mRNA expression levels of human *DNAH6* and mouse *Dnah6* were normalized to those of the actin gene (*ACTB* and *Actb*, respectively) using the following forward and reverse primers: *DNAH6*, 5′-CTAGAGCCTTTGCCAGTGCTA-3′ and 5′-AGGAGAGGGAGAGAACACCTC-3′; *ACTB*, 5′-CCTGGCACCCAGCACAAT-3′ and 5′-GGGCCGGACTCGTCATAC-3′; *Dnah6*, 5′-CTGGAGCCTCTGCCAGTGCTA-3′ and 5′-CGGAGAAGGAGAGAACACATC-3′; and *Actb*, 5′-AGATCAAGATCATTGCTCCTCC-3′ and 5′-AGCTCAGTAACAGTCCGCCT-3′. PCR conditions were as follows: 95 °C for 10 min; 34 cycles of 95 °C for 30 s, 57 °C for 30 s, and 72 °C for 30 s; and 72 °C for 10 min. The assay was performed three times.

### Immunofluorescence analysis

Sperm specimens were fixed in 4% paraformaldehyde for 30 min and permeabilized with 0.5% Triton X-100 for 10 min. Non-specific sites were blocked with 10% normal goat serum and 3% bovine serum albumin in phosphate-buffered saline, and the samples were incubated overnight at 4 °C with monoclonal anti-acetylated α-tubulin (T5168, 1:800), SPAG6 (HPA038440, 1:400), polyclonal anti- RSPH1 (HPA017382, 1:400), and polyclonal anti-AKAP4 (HPA020046, 1:200) antibodies (all from Sigma-Aldrich, St. Louis, MO, USA); and polyclonal antibodies against DNAH6 (ab122333, 1:50) and DNAH1 (ab122367, 1:100) (both from Abcam, Cambridge, UK). Alexa Fluor 488 anti-mouse (A-21121, 1:300) and Alexa Fluor 555 anti-rabbit (A31572, 1:300 dilution) IgG (both from Life Technologies, Carlsbad, CA, USA) were used as secondary antibodies. Specimens were counterstained with 4′,6-diamidino-2-phenylindole for 5 min, and fluorescence signals were visualized with a BX-51 fluorescence microscope (Olympus, Tokyo, Japan). Images were captured using VideoTesT-FISH v.2.0 software (VideoTesT, St. Petersburg, Russia).

## Supplementary information

Supplement information
