# Supplementary material for: Identification of DNAH6 mutations in infertile men with multiple morphological abnormalities of the sperm flagella
Source: Sci Rep. 2019 Nov 1;9:15864. doi: 10.1038/s41598-019-52436-7 (PMC6825154; doi:10.1038/s41598-019-52436-7)
Supplement: Supplementary file 1 — Supplement information [file 41598_2019_52436_MOESM1_ESM.docx]

**Supplement information**

**Identification of *DNAH6* mutations in infertile men with multiple morphological abnormalities of the sperm flagella**

Chaofeng Tu^1,2,3†^, Hongchuan Nie^1,2,3†^, Lanlan Meng^2^, Shimin Yuan^2^, Wenbin He^1,2,3^, Aixiang Luo^1^, Haiyu Li^1^, Wen Li^1,2,3^, Juan Du^1,2,3^, Guangxiu Lu^1,2,3^, Ge Lin^1,2,3^, Yue-Qiu Tan^1,2,3,^*

**Supplement Figure**

**
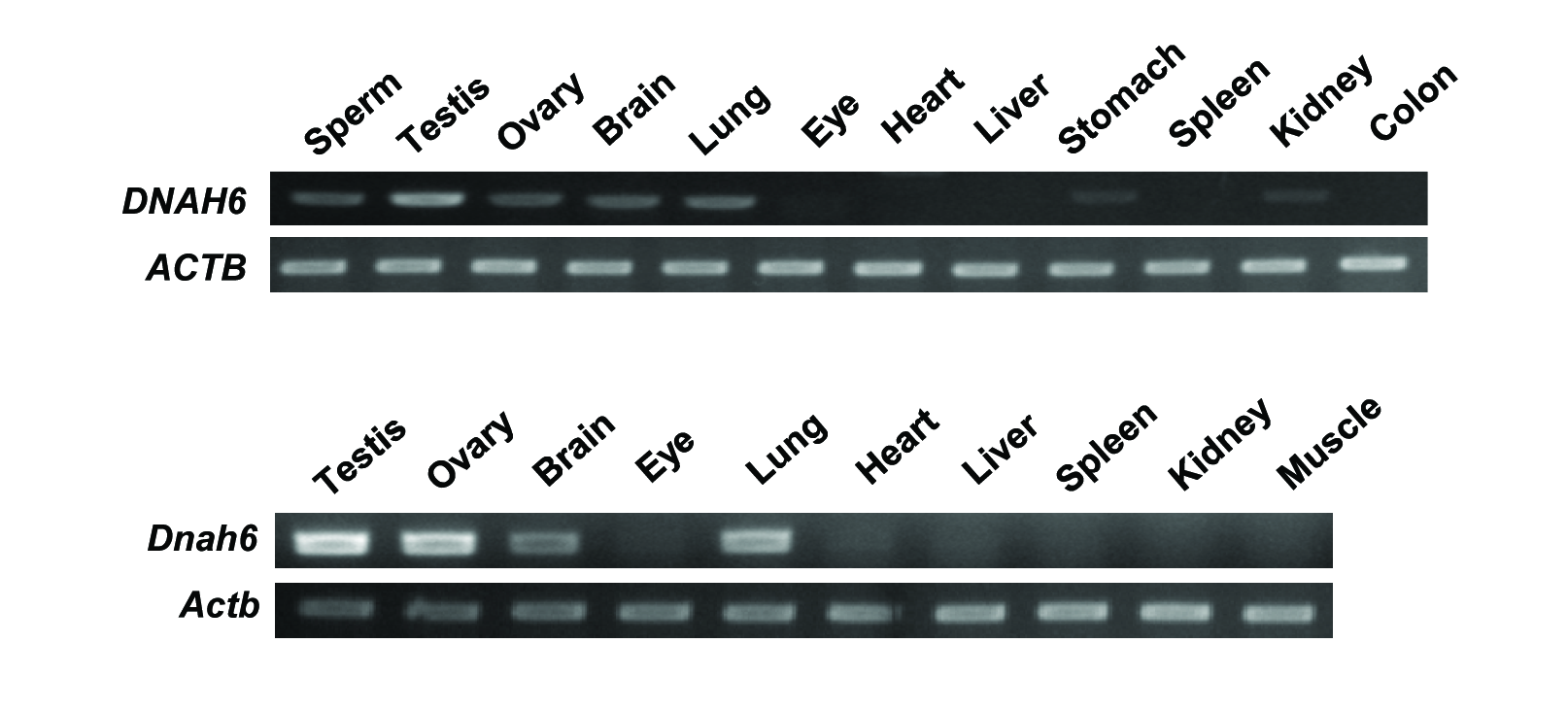
**

**Supplement Figure 1. Expression profiles of *DNAH6*.** Expression patterns of *DNAH6* in both human and mouse adult tissue were evaluated by reverse transcription PCR.

**Supplement tables**

**Supplement Table 1 Summary of whole exome sequencing data from the proband (P1).**

| **Sample ID** | **P1** |
| --- | --- |
| Bases in target region(bp) | 50621019 |
| Capture specificity (%) | 88.8051 |
| Bases mapped to genome(Mb) | 12799.65 |
| Reads mapped to genome | 85602441 |
| Reads uniquely mapped to genome | 30962355 |
| Reads mapped to target regions | 68158537 |
| Reads uniquely mapped to target regions | 27496157 |
| Mean depth of target region (X) | 159.28 |
| Coverage of target region (%) | 99.79 |
| Fraction of target region covered >=4X (%) | 99.57 |
| Fraction of target region covered >=10X (%) | 99.05 |
| Fraction of target region covered >=20X (%) | 97.78 |
| Mean depth of chrX (X) | 88.18 |
| Mean depth of chrY (X) | 77.98 |
| Duplication rate (%) | 26 |
| GC (%) | 50.18 |
| Gender test result | Male |

**Supplement Table 2 Summary of SNPs and Indels for the sample of the proband (P1).**

| **Sample ID** | **P1** |
| --- | --- |
| **Total SNPs** | **107317** |
| Heterozygous SNPs | 58476 |
| Homozygous SNPs | 48841 |
| Synonymous variant | 10730 |
| Missense variant | 10850 |
| Stop gained | 148 |
| Stop lost | 68 |
| Splice region variant | 2072 |
| Intron variant | 64101 |
| **Total INDELs** | **20020** |
| Heterozygous INDELs | 12098 |
| Homozygous INDELs | 7922 |
| Frameshift variant | 504 |
| Inframe-insertion | 190 |
| Inframe-deletion | 209 |
| Stop gained | 6 |
| Splice region variant | 583 |
| Intron variant | 14759 |

Note: (1) For SNPs, stopgain means that a nonsynonymous SNV that lead to the immediate creation of stop codon at the variant site. Meanwhile stoploss means that lead to the immediate elimination of stop codon at the variant site.
(2) Splicing is defined as variant that is within 2-bp away from an exon/intron boundary.
(3) Frameshift mutation means that an insertion/deletion of one or more nucleotides that cause frameshift changes in protein coding sequence.
(4) inframeshift mutation means that an insertion/deletion of 3 or multiples of 3 nucleotides that do not cause frameshift changes in protein coding sequence.
(5) For Indels, stopgain means that a frameshift insertion/deletion, nonframeshift
insertion/deletion or block substitution that lead to the immediate creation of stop codon at the variant site.

(6) For frameshift mutations, the creation of stop codon downstream of the variant will not be counted as "stopgain". Meanwhile stoploss means that lead to the immediate elimination of stop codon at the variant site.

**Supplement Table 3 Screening and identifying the causal genes by WES.**

| **Screening procedure** | **P1** |
| --- | --- |
| Total SNPs/IDNELs | 107317/20020 |
| Filter rare variants (MAF<5%)  in four public databases | 18881 |
| Predicted to be deleterious ^1^ | 362 |
| Homozygous or  compound heterozygous | 5/8 |
| Relevancy for phenotype^2^ | 1 |

**Note:** ^1^ represent variants predicted to be deleterious or loss of function; ^2^ represent variants associated with phenotype, such as expression, biological process, model organisms with the male sterility phenotype similar to the one observed in this family.

**Supplement Table 4 Candidate homozygous or compound heterozygous variants for the proband (P1).**

|  | **Gene** | **RefSeq ID** | **Chr** | **Pos** | **AAChange** | **Function** |
| --- | --- | --- | --- | --- | --- | --- |
| Homozygous  (F1) | ULK4 | NM_001322501 | 3 | 41942255 | c.T343C:p.S115P,ULK4 | missense |
|  | CEP162 | NM_001286206 | 6 | 84903394 | c.G814C:p.E272Q | missense |
|  | TBXAS1 | NM_030984 | 7 | 1.4E+08 | c.C1372T:p.R458C | missense |
|  | ZNF626 | NM_001076675 | 19 | 20807133 | c.1383_1549del:p.A461fs | frameshift |
|  | RBMXL3 | NM_001145346 | X | 1.14E+08 | c.G2825A:p.R942H | missense |
| Compound heterozygous  (F1) | FLG | NM_002016 | 1 | 152278768 | c.8593_8594insGG:p.A2865fs | frameshift |
|  | FLG | NM_002016 | 1 | 152278770 | c.8590_8591del:p.H2864fs | frameshift |
|  | **DNAH6** | **NM_001370** | **2** | **84899578** | **c.C6582A:p.D2194E** | **missense** |
|  | **DNAH6** | **NM_001370** | **2** | **85023448** | **c.G11258A:p.G3753D** | **missense** |
|  | ZNF141 | NM_003441 | 4 | 338197 | c.204_205insGA:p.H68fs | frameshift |
|  | ZNF141 | NM_003441 | 4 | 338201 | c.209_210del:p.I70fs | frameshift |
|  | MAML3 | NM_018717 | 4 | 140811063 | c.1513_1514del:p.Q505fs | frameshift |
|  | MAML3 | NM_018717 | 4 | 140811085 | c.1494_1504del:p.Q498fs | frameshift |
|  | FAM149B1 | NM_173348 | 10 | 74937720 | c.C269T:p.S90F | missense |
|  | FAM149B1 | NM_173348 | 10 | 74968403 | c.570dupT:p.H190fs | frameshift |
|  | MUC6 | NM_005961 | 11 | 1018215 | c.4585_4586insTG:p.T1529fs | frameshift |
|  | MUC6 | NM_005961 | 11 | 1018222 | c.4577_4578del:p.H1526fs | frameshift |
|  | KIR2DL1 | NM_014218 | 19 | 55286772 | c.527_528del:p.K176fs | frameshift |
|  | KIR2DL1 | NM_014218 | 19 | 55286775 | c.529_530insCA:p.V177fs | frameshift |
|  | SLC25A5 | NM_001152 | X | 118603873 | c.G361T:p.G121C | missense |
|  | SLC25A5 | NM_001152 | X | 118603925 | c.G413A:p.R138H | missense |

**Table S5 Candidate variants matching the screening criteria in ten families.**

| **Patients** | **Gene** | **Location** | **Amino Acid Alteration** | **dbSNP**  **ID** | **ExAC** | **1000 G** | **GO-**  **ESP** | **IN-**  **HOUSE** | **Mutation**  **Taster** | **SIFT** | **PolyPhen** | **CADD** |
| --- | --- | --- | --- | --- | --- | --- | --- | --- | --- | --- | --- | --- |
| P1 and P2 | *DNAH6* | Chr2:  84899578 | c.6582C>A  p.Asp2194Glu | - | - | - | - | - | disease causing | Deleterious  (0) | probably_  damaging(0.99) | 19.82 |
| P1 and P2 | *DNAH6* | Chr2:  85023448 | c.11258G>A p.Gly3753Asp | - | - | - | - | - | disease causing | Deleterious  (0) | probably_  damaging(1) | 26.5 |
| P3 | *DNAH6* | Chr2:  84822867 | c.2823dupT  p.S941fs | - | - | - | - | - | - | - | - | - |
| P3 | *DNAH6* | Chr2:  84954845 | c.10025G>A  p.Arg3342His | rs188849403 | 0.000138 | 0.001 | - | - | disease causing | Deleterious  (0.03) | probably_  damaging(0.969) | 19.74 |
| P5 | *DNAH1* | Chr3: 52397021 | c.G5105C:  p.R1702P | - | - | - | - | - | disease causing | Deleterious  (0) | probably_  damaging(0.99) | 27.2 |
| P5 | *DNAH1* | Chr3: 52430998 | c.11726_11727del:  p.P3909fs | - | 0.00008 | - | - | - | - | - | - | - |
| P7 | *DNAH1* | Chr3: 52417875 | c.8151-1G>C | - | - | - | - | - | - | - | - | 21.8 |
| P7 | *DNAH1* | Chr3: 52433062 | c.C12286T:p.R4096C | - | 0.00002 | - | 0.00008 | - | disease causing | Deleterious  (0) | probably_  damaging(1) | 26 |

Note: IN-HOUSE is a database including 5000 WES data of Chinese individuals obtained from Beijing Genomics Institute (BGI, China). Mutation Taster: the probability value refers to the prediction, i.e. a value close to 1 indicates a high 'security' of the prediction. SIFT score ranges from 0 to 1. Amino acid substitution is predicted damaging if the score is ≤ 0.05 and tolerated if the score is ≥ 0.05. PloyPhen-2 score: ranges from 0 to 1, and 0 is benign and 1 is damaging. CADD score: amino acid substitution is predicted damaging if the score is >15.
